# The effects of transcranial direct current stimulation and fluency shaping techniques on enhancing speech fluency in adults who stutter: Protocol for a double-blind randomized controlled trial

**DOI:** 10.1016/j.conctc.2025.101534

**Published:** 2025-08-07

**Authors:** Kowsar Esfandeh, Ahmad Reza Khatoonabadi, Hamid Karimi, Michael Nitsche, Abbas Ebadi

**Affiliations:** a Department of Speech Therapy, School of Rehabilitation Sciences, Tehran University of Medical Sciences, Tehran, Iran; b Department of Speech Therapy, School of Rehabilitation Sciences, Hamadan University of Medical Sciences, Hamadan, Iran; c Department of Geriatric Health, School of Rehabilitation Sciences, Tehran University of Medical Sciences, Tehran, Iran; d Endocrinology and Metabolism Research Institute, Endocrine Population Sciences Institute, Elderly Health Research Center, Tehran, Iran; e Faculty of Health, Charles Darwin University, Darwin, Australia; f Department of Psychology and Neurosciences, Leibniz Research Centre for Working Environment and Human Factors (IfADo), Dortmund, Germany; g Bielefeld University, University Hospital OWL, Protestant Hospital of Bethel Foundation, University Clinic of Psychiatry Psychotherapy, Bielefeld, Germany; h German Center for Mental Health (DZPG), Bochum, Germany; i Nursing Care Research Center, Clinical Sciences Institute, Baqiyatallah University of Medical Sciences, Tehran, Iran

**Keywords:** Speech fluency, Fluency shaping, Transcranial direct current stimulation, Stuttering, Adults who stutter

## Abstract

**Background:**

Stuttering is a condition that usually begins in childhood and may continue into adulthood. In this study, it is suggested that the combined approach of transcranial direct current stimulation (tDCS) with fluency shaping techniques will result in greater improvements in speech fluency compared to using fluency shaping techniques alone.

**Methods:**

This study is designed as a randomized, double-blind, sham-controlled clinical trial. All participants will participate in speech therapy sessions. The intervention group will receive anodal transcranial direct current stimulation, while the control group will experience sham stimulation. Participants will be randomly assigned to one of these groups. Before starting the treatment program, a preintervention assessment will be conducted to determine the severity of stuttering. Once these assessments are completed, each participant will take part in intervention sessions. Post-intervention evaluations will take place immediately after and six weeks after the final intervention session. Additionally, to evaluate the long-term stability of the treatment results, follow-up evaluations will be carried out three months post-treatment. The primary outcome measure—the ratio of stuttered syllables—will be evaluated during pre-, post-, and follow-up assessments; secondary outcomes will encompass scores from the Overall Assessment of the Speaker's Experience of Stuttering, severity ratings, and evaluations of speech naturalness.

**Conclusions:**

The results of this study will provide evidence regarding the effectiveness of combining tDCS with fluency shaping intervention on stuttering. This effect is likely to involve not only a reduction in SS% but also improvements in the overall quality of life for adults who stutter.

**Trial registration:**

IRCT20230907059369N1.

## Highlights

• This study aims to examine the effects of anodal transcranial direct current stimulation (tDCS) on enhancing speech fluency in adults who stutter.
• A double-blind, randomized controlled trial will be conducted to measure changes in stuttering severity following anodal transcranial direct current stimulation.
• Sixty-two adults who stutter will be recruited to receive either anodal or sham transcranial direct current stimulation over the inferior frontal gyrus, combined with fluency shaping techniques.

## 1 Introduction

Stuttering is a neurodevelopmental disorder that affects speech fluency. It is characterized by interruptions in the natural rhythm and flow of speech production [1]. It involves involuntary repetitions and prolongations of speech sounds and syllables, and temporary interruptions or blocks in the natural flow of speech. Although the natural recovery rate is high during the initial three years after the onset of stuttering, around 1 % of adults globally continue to experience chronic stuttering [2]. Stuttering can impact various aspects of an individual's life. People who stutter(PWS) report experiencing negative reactions from others, difficulties communicating in important situations, lower levels of life satisfaction, and reduced ability to achieve their life goals [3]. Stuttering is classified as a “multifactorial” disorder involving genetic factors and neurological anomalies. However, there is a consensus among researchers that it is neurological in nature [4].

Although multiple therapeutic approaches have been developed in conjunction with various theoretical paradigms regarding the nature and etiology of stuttering, behavioural stuttering treatments have demonstrated the highest level of evidence [[5], [6], [7]]. However, some previous treatments reported changes in neurological functions of people who stutter after behavioural treatments [[8], [9], [10], [11]]. In these treatments, the individual learns a new speech pattern that requires extensive practice and time for generalization to everyday situations. The implementation of this new pattern may sometimes affect the naturalness of the individual's speech, which consequently reduces acceptance of the new method [12]. Behavioral “fluency shaping” therapies, which aim to modify factors like speech tempo, prosody, rhythm, speech onsets, and breathing techniques, have proven effective in significantly reducing the severity of stuttering, achieving less than 1 % of stuttered syllables [10]. Fluency-shaping therapies have been shown to reduce over-activation observed in the right hemisphere, normalize activity patterns in the basal ganglia, and reactivate the left-hemispheric cortical regions [[9], [10], [11],^13^,^14^].

Enhanced fluency is furthermore accompanied by increased functional connectivity within the sensorimotor integration network. In particular, two specific connections display strengthening; the left inferior frontal gyrus shows enhanced connectivity with the precentral gyrus at the site of the left laryngeal motor cortex, and the left inferior frontal gyrus demonstrates heightened connectivity with the right superior temporal gyrus. Consequently, the neural remediation linked with therapy is based on a strengthened integration of the command-to-execution pathway in conjunction with an enhanced auditory-to-motor coupling [15,16]. Nevertheless, maintaining therapeutic gains requires ongoing practice and training, as sustained improvements in fluency remain challenging to achieve without continuous intervention, Therefore, the relateralization of the speech-related neural network is commonly only a temporary effect, and represents an ultimately inadequate repair process overall [10]. As a result, there is an ongoing need to explore and develop novel therapeutic approaches to more effectively improve or alleviate stuttering symptoms [4].

Recent research has proposed integrating neuromodulation techniques with traditional therapeutic interventions. Neuromodulation has demonstrated the capacity to enhance brain function through the application of NIBS techniques. Non-invasive brain stimulation provides a means for direct interaction with the mechanisms of neural tissue. Consequently, it has been used to gather further and ‘real-time’ understanding of the impaired or abnormal motor functions that characterize developmental stuttering. In general, NIBS techniques can effectively alter neural functioning patterns through modulation of brain network activity [4]. One of the non-invasive brain stimulation methods that has been designed and used for the treatment of brain disorders is transcranial direct current stimulation (tDCS) [17].

In tDCS, an electrical current is applied between two or more electrodes, which include a negatively charged cathode and a positively charged anode, strategically positioned on the scalp. The administration of this current (with an intensity of 1–2 mA) induces minor modifications to the resting membrane potential of cortical neurons located within the surrounding brain tissue [17]. Specifically, within the framework of established protocols, cathodal stimulation affects neuronal compartments by inducing hyperpolarization, which leads to a decrease in neuronal excitability. In contrast, anodal stimulation causes depolarization, resulting in an overall increase in excitability at the macroscale level [[18], [19], [20], [21]].

Previous evidence indicates that the combination of non-invasive brain stimulation and behavioral interventions results in greater improvements [4]. Findings from these studies have prompted researchers to explore the utilization of tDCS as an adjunctive method in the treatment of speech fluency disorders in adults who stutter. The application of brain stimulation techniques for the treatment of stuttering in adults who stutter (AWS) is a novel approach, with comparatively limited research conducted thus far to other neurogenic communication disorders, such as aphasia or apraxia of speech. Recent investigations have shown that tDCS facilitates speech fluency in adults who stutter when combined with a fluency intervention [[22], [23], [24], [25], [26], [27]]. Recent findings indicate that abnormalities in the frontal cortex are present in individuals who stutter [28], identifying this region as a potential target for tDCS, which has been explored in recent studies [22,23,25]. Recently, a limited number of studies have explored the application of tDCS in conjunction with speech restructuring techniques that incorporate external timing cues (such as choral speech and metronome-timed speech) for the intervention of stuttering in AWS [[22], [23], [24], [25]].

This study further investigated the efficacy of tDCS for reducing observable and covert stuttering behaviors with more number of stimulation sessions and dosage, and while person who stutter control their fluency using the techniques that they have learned during the treatment. In previous studies, participants experienced fluent speech during brain stimulation with the external helps (e.g., unison speech, delayed auditory feedback (DAF), or metronome pacing) [[22], [23], [24], [25], [26]], However, in this study, participants be trained to developed self-control skills using fluency-shaping techniques during brain stimulation [29,30]. Additionally, tasks during brain stimulation in earlier studies were limited to reading sentences, passages, or monologues [[22], [23], [24], [25], [26], [27]], In contrast, in this study we stimulated the brain while the person performed various tasks such as reading, monologues, dialogues with the clician, dialogues with strangers, and telephone conversations with friends and strangers. This approach is based on findings suggesting that PWS encounter greater difficulties in maintaining fluent speech during cognitively demanding conversational tasks, as well as increased challenges in sustaining fluency when engaged in complex speech tasks that require syntactic or linguistic processing [[31], [32], [33]]. Also, AWS experience heightened difficulties in sustaining speech fluency during complex social interactions compared to private speech contexts, primarily due to increased anticipation and social pressures [34,35]. Furthermore, stimulation dosage in this study will be increased compared to previous studies, which typically used intensities of 1, 1.5, or 2 mA in 20-min sessions per day [[22], [23], [24], [25], [26], [27]]. This adjustment will be based on research suggesting that increased stimulation duration and intensity will not cause adverse effects, even in children, and will enhance the efficacy of treatment. Additionally, trends and suggestions in other areas of speech pathology will be explored [[36], [37], [38]].

The aim of this research will be to investigate the effects of adjunctive non-invasive brain stimulation on speech fluency in adults with stuttering. To accomplish this goal, two groups of adult participants who stutter will be recruited, namely a sham tDCS group and an anodal tDCS group. In the sham tDCS group, participants will receive sham stimulation simultaneously with the fluency shaping intervention, whereas those in the anodal tDCS group will undergo anodal tDCS in conjunction with the fluency intervention. It will be hypothesized that combining the fluency intervention with anodal tDCS aimed at the left IFG will produce more favorable outcomes than the sham tDCS control condition in improving speech fluency among adults who stutter.

## 2 Methods

### 2.1 Study design

This study is structured as a randomized, double-blind, sham-controlled clinical trial that will be performed to assess the efficacy of anodal tDCS in conjunction with a fluency shaping intervention focused on enhancing speech fluency in adults who stutter. Participants will be randomly assigned to either the control group or the intervention group. In the anodal tDCS group, participants will undergo fluency shaping intervention alongside anodal tDCS, while the sham tDCS group will receive sham tDCS concurrently with the fluency shaping intervention. The study will be conducted at the Speech Therapy Clinic and tDcs lab, School of Rehabilitation, Tehran University of Medical Sciences. The findings will be reported based on the CONSORT (Consolidated Standards of Reporting Trials) guidelines [39]. This trial has been registered at the Iranian Registry of Clinical Trials (IRCT code: IRCT20230907059369N1), where detailed information about the study protocol, inclusion and exclusion criteria, interventions, outcomes, and ethical approval can be found. The trial registration process was completed before the enrollment of the first participant. The current protocol is reported according to the Standard Protocol Items: Recommendations for Interventional Trials (SPIRIT) guideline [40].

Data will be gathered at four different time points: one week before the intervention, immediately after, 6 weeks after, and 3 months after the intervention. We will examine the effectiveness of anodal tDCS combined with fluency shaping techniques in improving speech fluency by comparing the outcomes of the different treatments. In pre-treatment assessments, we will measure primary and secondary outcome measures before treatment. Pre-treatment assessments will be conducted to evaluate the severity of stuttering before the treatment program begins. The primary outcome measure will be the Percentage of Stuttered Syllables (%SS) obtained from an unscheduled telephone call. Secondary outcome measures will include Severity Rating (SR) and Speech Naturalness obtained from an unscheduled telephone call, and the Overall Assessment of the Speaker's Experience of Stuttering (OASES). After completing these assessments, each participant will engage in treatment sessions. Post-treatment assessments will be conducted immediately after, 6 weeks after, and 3 months after the treatment to evaluate changes in primary and secondary outcome measures and to assess the maintenance and long-term stability of the results.

**Fig. 1:**
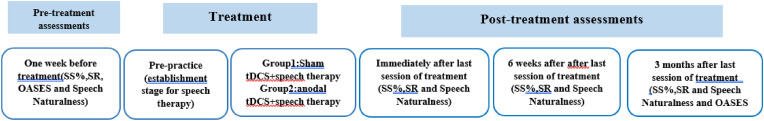
Trial design and timeline. OASES: Overall Assessment of the Speaker's Experience of Stuttering; SS%:percentage of stuttered syllables; SR:Severity Rating; tDCS: transcranial direct current stimulation.

The study will focus on adults with moderate-to-severe stuttering. Therefore, stuttering severity will be evaluated before recruitment to confirm enrollment eligibility. The severity of stuttering will be assessed by calculating SS% in unscheduled telephone call for each participant. At the baseline assessment, a 10-min unscheduled telephone call with each participant will be conducted by individuals other than the researcher and this call will be recorded using a call recorder application. Using these data, the primary outcome measure (SS%) will be calculated. Two experienced specialist raters will independently evaluate and calculate the SS% of all speech samples. To assess interrater reliability, Cohen's kappa will be calculated. If the raters agree (intraclass correlation coefficient between 0.8 and 1), the SS% will be reported. However, if there is disagreement (intraclass correlation coefficient below 0.8), a third senior rater will review and evaluate the SS%. The completion of the OASES questionnaire by participants constitutes the final step of the pre-treatment assessments. Following the baseline assessments, the speech therapy sessions will begin. Three time points after the final intervention session (i.e., immediately, six weeks, and three months), each participant will complete post-treatment assessments, which will be identical to the pre-treatment assessments and conducted by the same raters. The trial's design and timeline are depicted in Fig. 1.

### 2.2 Participants and eligibility criteria

The study population will include adults with moderate-to-severe developmental stuttering who have not received any treatment for at least one month before the intervention. Adults aged between 18 and 50 who stutter will be recruited for this research. The speech-language pathologist will evaluate each participant's stuttering severity to determine if they qualify for enrollment in the study. Inclusion criteria will include a history of developmental stuttering, diagnosis of moderate-to-severe stuttering, right-handedness, age between 18 and 50 years, native speaker of Farsi, no other speech or language disorders other than stuttering, no history of hearing loss, neurological or psychiatric disorders, or seizures, and no intake of any medication that affects brain functions, such as antidepressants. Additionally, individuals who are pregnant, breastfeeding, or have cranial bone defects, cranial or brain metal implants, or skin lesions will be excluded from this study.

### 2.3 Interventions

The treatment will have two stages. Stage 1:Speech therapy (Pre-practice or Establishment stage) and Stage 2: transcranial Direct Current Stimulation intervention(Practice stage).

#### 2.3.1 Speech therapy (pre-practice or establishment stage)

Both the control and intervention groups will undergo this treatment stage before the start of the tDCS intervention, which will be referred to as the pre-practice(establishment stage). In this stage, the clinician will work with the participant to practice fluency shaping techniques, progressing from simple to complex levels. Each participant will engage in 1–2 h intensive sessions at the clinic to practice fluency-shaping techniques (such as easy onset, airflow, pausing, light consonant contact, and prolongation) at basic levels (phonemes, syllables, words, sentences, and phrases) with the clinician. Subsequently, more complex tasks will be introduced, including reading, monologues, dialogues with the clinician, dialogues and monologues in the presence of strangers, and practicing telephone calls. The goal of this stage will be to ensure that the participant can use fluency shaping techniques in various speech tasks with the clinician in the clinic before entering the combined intervention stage (fluency shaping techniques and brain stimulation). The study participants will be expected to maintain a severity rating (SR) below 2 during these tasks before proceeding to the practice stage. This stage will ensure that clients can use the restructured speech techniques at a simple level with the clinician at the clinic. Then, clients will use these techniques during more complex online practice tasks along with anodal tDCS/Sham tDCS.

#### 2.3.2 Transcranial current direct stimulation Intervention(Practice stage)

In this stage of treatment, participants will be randomly allocated to two groups: the intervention group (anodal tDCS) and the control group (sham tDCS). Clients will practice fluency shaping techniques as an online treatment alongside each of ten 20-min tDCS stimulations. Clients will start by reading simple sentences and monologues during the first tDCS session but will eventually progress to more challenging tasks, such as conversing with the clinician, a stranger at the clinic, making telephone calls, and performing dual tasks. The tasks will become hierarchically more complex linguistically and cognitively.

In the tDCS protocol, electrical stimulation will be administered by applying a 2 mA current through two electrodes measuring 5 cm × 7 cm. The process will last for 40 min, with 15-s ramp-up and ramp-down intervals. Each therapy session will last 60 min (20 min of stimulation, 20 min of rest, followed by another 20 min of stimulation). Accordingly, each participant will receive 10 stimulation sessions over 5 therapy sessions conducted on 5 consecutive days. The tDCS will be administered using a Starstim DC stimulator. The tDCS electrodes will consist of conductive rubber encased in sponge pockets, with saline solution used as the electrolyte-based contact medium. It will be essential to saturate the sponges thoroughly with saline. The sponges will need to be properly moistened on all sides without becoming excessively saturated to avoid any dripping. Before placing the electrodes on the scalp, the clinician will examine the skin for any damage or lesions. The same stimulation intensity parameters will be applied for both anodal and sham modes. However, in the sham mode, the device will automatically reduce the intensity after 30 s. An electroencephalogram (EEG) cap will be utilized to determine the stimulation site. The anode electrode will be positioned over the left frontal gyrus (FC5, based on the 10–20 international system) [41], while the cathode electrode will be placed over the right frontopolar region (FP2, according to the same system) [41]. Elastic rubber straps will be used to secure the electrodes in place and prevent any movement.

### 2.4 Study outcome measures

#### 2.4.1 Primary outcome measures

In this research, the average scores of the percentage of Stuttered Syllables (%SS) obtained from a 10-min unscheduled telephone call will serve as the primary outcome measure. A baseline assessment will be collected one week before the intervention. The identical assessment will be conducted immediately after, and six weeks and three months following the last intervention session (post-treatment assessments). A 10-min unscheduled telephone call with each participant will be conducted by individuals other than the researcher, and this call will be recorded using a call recorder application. Two experienced specialist raters, blinded to the treatment conditions and assessment time points, will independently evaluate and calculate the %SS of all speech samples. The recordings will be coded and screened to ensure anonymity. To calculate the %SS, the total number of syllables associated with unambiguous stuttering for each recorded sample will be counted, divided by the total number of syllables for that sample, and then multiplied by 100 [42]. Stuttering will be characterized as occurrences that involve either (i) repetitions of phonemes, words, or phrases, (ii) prolonged phonemes, or (iii) blocks. It will be important to highlight that, per Riley's definition of stuttering (1994), interjections, silent blocks, and repetitions of whole words or phrases will be considered stuttering if they are articulated abnormally and with tension. Otherwise, they will not meet the criteria for stuttering [43]. The expected mean %SS score at the three post-intervention time points in the anodal tDCS group will be hypothesized to be significantly lower than that found in the sham tDCS group (a diminished %SS score will indicate a less severe form of stuttering). Additionally, no significant difference will be expected between groups at the baseline time point.

#### 2.4.2 Secondary outcome measures

Secondary outcome measures will include (i) the overall score for severity rating (SR), which will provide an overall judgment of the stuttering severity in speech, (ii) the overall score for speech naturalness [44], and (iii) the overall score from the OASES questionnaire [45], which will review the psycho-social consequences of stuttering from the viewpoint of the individual who stutters.

##### 2.4.2.1 Severity Rating(SR)

Severity Rating is a perceptual evaluation of stuttering, providing an overall judgment of the stuttering severity in speech [44]. In this study, the severity of stuttering will be rated using a 10-point scale, where a score of 0 indicates no stuttering, a score of 1 represents very mild stuttering, and a score of 9 indicates very severe stuttering.

##### 2.4.2.2 Speech naturalness

During speech fluency shaping therapy, clients are trained to use an unnatural speech pattern to control their stuttering. Throughout the therapy, they learn to make their speech sound more natural. Essentially, clients need to understand how natural their speech appears to others [46]. In this study, the naturalness of speech will be assessed using a 9-point scale, where a score of 1 indicates the absence of any fluency-shaping techniques, and a score of 9 reflects an exaggerated use of these techniques [44].

##### 2.4.2.3 Overall Assessment of the Speaker's experience of Stuttering(OASES) questionnaire

In this study, stuttering severity will be assessed using the OASES questionnaire. This tool will evaluate the individual's experience of stuttering across four sections: general information about stuttering, reactions to stuttering, communication in daily situations, and quality of life. Participants will respond to questions using a 5-point scale. The test results will be reported in two values: Impact Scores and Impact Rating, which will reflect the effect and extent of stuttering on the individual's life. The Impact Scores will always range from 20 to 100, and based on this score, the Impact Rating will be classified from mild to severe [45]. In this study, the OASES questionnaire will be completed by participants both before the intervention to assess their comprehensive experience of stuttering and 3 months after the intervention to evaluate the sustainability of treatment effects on their stuttering experience and quality of life.

### 2.5 Randomization

This study is a randomized, double-blind, sham-controlled trial, where participants will be randomly assigned to either the intervention group or the control group. The random allocation of participants will help prevent selection bias and ensure a balanced distribution of confounding factors between the study groups. For the randomization process, blocked randomization will be employed. Specifically, one of the six blocks used in this study (ABAB, BABA, AABB, BBAA, BAAB, ABBA) will be randomly selected. A will represent the intervention group, and B will represent the control group. Each participant will be assigned an ID number and allocated to one of the treatment groups. The individual who generates the random allocation will not be involved in any other phase of the trial.

### 2.6 Blinding

As the study is double-blind, neither the participants nor the researcher will know of the group assignment for each participant. Sealed, opaque envelopes will be used to ensure concealment. A separate envelope will be created for each participant, labeled with an ID number, and the corresponding treatment group for each ID will be placed in its designated envelope. To ensure double blinding, the actual names of the treatment groups (intervention and control) will be replaced with specific codes unknown to both the principal investigator and the participants. A clinician, independent of the principal investigator, will assign these codes to the corresponding treatment groups. Before the principal investigator begins treating a participant, the clinician will provide the participant with one of the sealed envelopes. After the participant opens the envelope, the clinician will configure the mode—either anodal or sham—according to the envelope's contents and attach the electrodes to the participant's head as described in the above sections. After the setup is completed, the principal investigator will initiate the treatment. Additionally, the researchers conducting the outcome assessments and data analysis will remain blinded to the group assignments.

### 2.7 Efficacy

We anticipate that combining tDCS with the intervention will amplify its impact on the outcome measures and improve the durability of the treatment-induced gains.

### 2.8 Safety

The stimulation parameters used in this study are not expected to pose any significant risks. A systematic review examining the adverse effects of tDCS across all published studies, including those involving vulnerable populations, found that side effects like tingling and itching under the electrodes were mild and resolved quickly after stimulation. No serious adverse effects have been reported to date. Therefore, it can be concluded that tDCS, when applied within the specified parameters, is a safe intervention.

### 2.9 Sample size

Based on the previous investigations conducted by Karsan et al. and Moien et al. [25,26], and employing G∗Power software [47], it is determined that 31 subjects per group (totaling 62 participants overall) are required to detect a statistically significant difference regarding a time × group interaction via a two-way mixed model analysis of variance (ANOVA). The time variable is designated as the within-subject factor (i.e., before the intervention and immediately, six weeks, and 3 months following the intervention), while the group is classified as the between-subject factor (i.e., anodal or sham tDCS). The dependent variable examined is the percentage of stuttered syllables (SS%). Type I (α) and type II (β) error rates are set at 0.05 and 0.20, respectively, with an effect size of 0.17.

### 2.10 Statistical analysis

Descriptive statistics will be calculated for demographic and baseline characteristics, in addition to primary and secondary outcomes for all participants engaged in the study. For each participant, summary statistics including mean, standard deviation, median, minimum, and maximum values will be documented for quantitative variables. Frequency tables will be employed to present the data of qualitative variables. Differences among demographic variables will be assessed using Student's t-tests for quantitative variables and chi-square tests for qualitative variables. To evaluate the effect of tDCS on the primary and secondary outcome measures, mixed-model ANOVAs will be used, with group (anodal vs. sham) as the between-subjects factor and time (baseline, immediately post-treatment, 6 weeks, and 3 months) as the within-subjects factor.

### 2.11 Ethical approval al and consent to participate

This study will adhere to ethical principles as well as national norms and standards for conducting medical research in Iran (approval ID: IR.TUMS.MEDECINE.REC.1402.318, September 5, 2023, Research Ethics Committees of the School of Medicine, Tehran University of Medical Sciences). Furthermore, the ethics committee will review and approve the informed consent form. Before initiating any aspect of the study, the principal investigator will secure written informed consent from all participants.

### 2.12 Consent for publication

The researchers will have the freedom to use the study results for educational and scientific purposes, but written approval from the study sponsor will be obtained before submitting the manuscript.

### 2.13 Availability of data and materials

All information about participants will be kept confidential and will not be made available to the general public. Additionally, every document, data set, audio recording, and other materials will be assigned a unique participant identification number for coding, ensuring that the participants' identities remain confidential. To ensure confidentiality, the names of the subjects will not be disclosed, and all related materials will be coded with participant identification numbers. The data sets utilized and assessed during the study will be obtainable from the corresponding author upon making a reasonable request.

## 3 Discussion

This study is a randomized controlled trial with follow-up assessments to evaluate the effectiveness of combining tDCS with fluency shaping intervention to enhance speech fluency in adults who stutter. The primary outcome measure to assess the treatment's impact will be the percentage of syllables stuttered (SS%). The intervention's effects will be monitored for up to 3 months to rule out any short-lived improvements with limited clinical relevance. The results of this study will provide valuable insights into the effectiveness of tDCS as a supplementary treatment for reducing the percentage of syllables stuttered (SS%) and its long-term stability over an extended period.

In this study, we will stimulate the IFG while the person performs various tasks such as reading, monologues, dialogues with the clinician, dialogues with strangers, and telephone conversations with friends and strangers. This approach considers findings that individuals with stuttering face greater challenges in maintaining fluency during complex conversational tasks [[31], [32], [33]]. Furthermore, we will assess the effect of stuttering on an individual's quality of life using the OASES questionnaire. This tool provides a thorough evaluation of stuttering's impact, measuring its influence on various life circumstances and the individual's overall well-being [45]. In conclusion, we expect this study to demonstrate that combining tDCS with fluency shaping intervention will have a supplementary effect on stuttering. This effect is likely to involve not only a reduction in SS% but also improvements in the overall quality of life for adults who stutter.

## 4 Conclusion

Based on the expected outcomes of the current study, non-invasive brain stimulation can serve as a complementary approach alongside a fluency enhancement intervention to enhance the effectiveness of the intervention and decrease its duration. Furthermore, a fluency intervention paired with tDCS has the potential to mitigate the adverse effects of living with stuttering for individuals who stutter. Given that brain stimulation through tDCS is cost-effective and user-friendly, it can be readily implemented by clinicians.

## CRediT authorship contribution statement

**Kowsar Esfandeh:** Writing – review & editing, Writing – original draft, Methodology, Conceptualization. **Ahmad Reza Khatoonabadi:** Writing – review & editing, Supervision, Funding acquisition. **Hamid Karimi:** Writing – review & editing, Writing – original draft, Methodology, Conceptualization. **Michael Nitsche:** Writing – review & editing, Methodology, Conceptualization. **Abbas Ebadi:** Writing – review & editing.

## Funding

This study was supported by the 10.13039/501100004484Tehran University of Medical Sciences (grant number: 1403-3-288-72825).

## Declaration of competing interest

The authors declare that they have no known competing financial interests or personal relationships that could have appeared to influence the work reported in this paper.

## Data Availability

No data was used for the research described in the article.
